# Delicate Balances in Cancer Chemotherapy: Modeling Immune Recruitment and Emergence of Systemic Drug Resistance

**DOI:** 10.3389/fimmu.2020.01376

**Published:** 2020-06-30

**Authors:** Anh Phong Tran, M. Ali Al-Radhawi, Irina Kareva, Junjie Wu, David J. Waxman, Eduardo D. Sontag

**Affiliations:** ^1^Department of Chemical Engineering, Northeastern University, Boston, MA, United States; ^2^Department of Electrical and Computer Engineering, Northeastern University, Boston, MA, United States; ^3^Mathematical and Computational Sciences Center, School of Human Evolution and Social Change, Arizona State University, Tempe, AZ, United States; ^4^Clinical Research Institute, Key Laboratory of Tumor Molecular Diagnosis and Individualized Medicine of Zhejiang Province, Zhejiang Provincial People's Hospital, People's Hospital of Hangzhou Medical College, Hangzhou, China; ^5^Department of Biology and Bioinformatics Program, Boston University, Boston, MA, United States; ^6^Department of Bioengineering, Northeastern University, Boston, MA, United States; ^7^Laboratory of Systems Pharmacology, Program in Therapeutic Science, Harvard Medical School, Boston, MA, United States

**Keywords:** metronomic chemotherapy, cyclophosphamide, mathematical modeling, immune recruitment, cancer resistance

## Abstract

Metronomic chemotherapy can drastically enhance immunogenic tumor cell death. However, the mechanisms responsible are still incompletely understood. Here, we develop a mathematical model to elucidate the underlying complex interactions between tumor growth, immune system activation, and therapy-mediated immunogenic cell death. Our model is conceptually simple, yet it provides a surprisingly excellent fit to empirical data obtained from a GL261 SCID mouse glioma model treated with cyclophosphamide on a metronomic schedule. The model includes terms representing immune recruitment as well as the emergence of drug resistance during prolonged metronomic treatments. Strikingly, a single fixed set of parameters, adjusted neither for individuals nor for drug schedule, recapitulates experimental data across various drug regimens remarkably well, including treatments administered at intervals ranging from 6 to 12 days. Additionally, the model predicts peak immune activation times, rediscovering experimental data that had not been used in parameter fitting or in model construction. Notably, the validated model suggests that immunostimulatory and immunosuppressive intermediates are responsible for the observed phenomena of resistance and immune cell recruitment, and thus for variation of responses with respect to different schedules of drug administration.

## 1. Introduction

Immune system involvement in cancer progression has been well-established, leading to increased efforts to harness the ability of the host immune system to fight off growing tumors (1). Standard of care chemotherapy regimens typically involve drug administration on a maximum tolerated dose (MTD) schedule (2). These regimens aim to target most cancer cells at once, but frequently lead to a reduction in tumor burden only in the short term (3) and often give rise to drug resistance (4, 5). One reason for this longer-term failure is that MTD-based treatment can cause collateral damage to the host immune system, thus diminishing its ability to target the tumor (3). An optimal treatment regimen should strike a balance between drug-induced tumor cell kill and damage to the immune system, allowing the two modes of cancer cell elimination to complement each other. Indeed, high frequency low dose drug administration, also known as metronomic chemotherapy, can in some cases strike the right balance and induce immunogenic cell death (ICD) in tumor tissue by selecting an appropriate choice of drug, dose, and time interval between treatments (6–16). Successful achievement of ICD-based therapeutic outcomes during anticancer therapy is dependent on complex interactions between the drug, the tumor, and the host immune system, the nature of which is still being uncovered (17).

To better understand the mechanisms whereby metronomic chemotherapy enables an anti-tumor immune response, it is important to understand how tumors are able to evade immunosurveillance in the first place (1). An important and well-studied evasion route is through the accumulation of mutations and epigenetic modifications that help avoid immunosurveillance (18, 19). However, our mathematical model focuses on network effects, in contrast to such (epi)genetic changes in tumors, demonstrating that even the described mechanistic interactions are sufficient to explain experimental observations.

### 1.1. Interaction Dynamics Between Tumors and the Immune System

The proposed model is phenomenological, its components representing the combined effects of a variety of immunostimulatory as well as immunosuppressive processes. For background, we next briefly discuss some of these immune-related processes, which involve modifications of the tumor microenvironment (TME). Such modifications include increased acidity resulting from altered nutrient metabolism (20, 21) and altered balance between cytotoxic and regulatory immunity through the recruitment by the tumor of immunosuppressive cells, such as regulatory T cells (Tregs) (22–26) and myeloid-derived suppressor cells (MDSCs) (27, 28).

Some examples of TME modifications are as follows. Macrophages with an M2 phenotype can produce high levels of TGF-β, IL-10, and vascular endothelial growth factor (VEGF), promoting tumor growth (29–32). In other cases, tumor-derived factors and gangliosides can alter dendritic cell (DC) phenotype leading to lower levels of CD80, CD86, CD40, and high indoleamine 2,3-dioxygenase expression that contributes to suppression of T cell immunity (33). Immunosurveillance can also be evaded through production of various immunosuppressive cytokines such as TGF-β that play an important role in suppressing macrophages and monocytes (34). Other factors such as tumor necrosis factor (TNF)-α, IL-1, IL-6, colony stimulating factor (CSF)-1, IL-8, IL-10, and type 1 interferons (INFs) can also contribute to cancer growth (35–39). Additionally, pro-angiogenic factors such as VEGF can inhibit differentiation of progenitors into DCs (40). IL-10 and TGF-β can also inhibit DC maturation. Ganglioside antigens can also suppress cytotoxic T-cells (CTLs) and dendritic cells (DCs) function (41). Immunosuppressive enzymes such as IDO, arginase, and inhibitor of nuclear factor kappa-B kinase (IKK)2 may also contribute to tumor progression via direct actions on tumor cell proliferation or through induction of T cell tolerance/suppression (42–44).

### 1.2. Network Effects of Chemotherapy Interventions

By targeting various components that regulate immune tolerance, cancer chemotherapy drugs, such as mitoxantrone, idarubicin, doxorubicin, and cyclophosphamide can induce immunogenic cancer cell death in addition to their direct cancer cell cytotoxic effects (10, 11, 45, 46). By using an optimized drug dose and schedule of administration, favorable immune responses can be achieved, including increases in macrophage recruitment and maturation (47), proliferation of NK cells, levels of IFN-γ (48), as well as elevated post-apoptotic release of the nuclear chromatin binding protein HMGB1, which can stimulate antigen presentation by DCs, helping CD8^+^ T cell activation (49, 50). In some cases, type-1 interferon signaling pathways are switched on, leading to host antitumor immunity activation (51–54). Immunosuppressive molecules, like CD31, CD46, CD47, are downregulated on dendritic cells by ICD drug treatment (55). Molecular chaperones such as HSP90 appear on the tumor cell surface, promoting DC maturation (56). These optimized drug administration conditions can also lead to transient lymphopenia, which upregulates repair mechanisms and can lead to a vast array of immunostimulatory outcomes, including enhanced T-cell activation, immune recruitment, DC differentiation, and maturation, as well as the release of large amounts of chemokines and cytokines (57, 58). Cytotoxic effects on immunoregulatory cells, such as MDSCs and Tregs, contribute to restoration of anti-tumor immunity by decreasing suppression of T-cells and NK cells (59). Other factors affecting immunogenicity include changes in MHC-1 molecules and tumor-specific antigens on the tumor cell surface (60), stress-induced expression of NK cell stimulatory ligands, and decreases in NK inhibitory ligands (61).

The ability of drugs to induce anti-tumor immune responses is not sufficient by itself to ensure a successful therapeutic response, as the effect on various compartments of the immune system, and thus on overall tumor burden can vary dramatically depending on dose, schedule, and tumor type. Scheduling and dosing of an ICD drug is of critical importance in instigating an immune response, which relates to the concept of “getting things just right” (17, 62). For instance, administration of cyclophosphamide on a 6-day repeating schedule (Q6D) at 140 mg/kg per dose, dramatically improves the therapeutic outcome for murine GL261 gliomas through immunomodulatory mechanisms (12, 13, 15). Other drug treatment schedules, however, do not result in the same efficacy. This loss of efficacy correlated with reversal of an initial anti-tumor immune response, despite ongoing ICD drug treatments (12). Intriguingly, cyclophosphamide treatment of Lewis lung carcinoma (LLC) and B16F10 tumor at the same dose and on the same Q6D schedule does not result in tumor regression or immune cell recruitment, despite the intrinsic sensitivity of these two tumor lines to activated cyclophosphamide (16).

It is clear that much remains to be understood about the underlying mechanisms of ICD action including the impact of chemotherapy dose and schedule on the many factors linked to the ICD response (17). Well-designed mathematical models, which can help elucidate the complex interplay between the various players, make these models an invaluable complementary tool to *in vivo* experimental results for designing better treatments.

### 1.3. Mathematical Modeling of Tumors and the Immune System

There is a rich tradition in utilizing mathematical biology to study cancer chemotherapy and the immune system. A large variety of models have been proposed, notably in the work of de Pillis and collaborators, who introduced a series of models that depict many of the interactions between chemotherapy and immunotherapy drugs, the immune system, and tumor progression (63, 64). Closely related to our topic, Ciccolini et al. (65) proposed a pharmacokinetics and pharmacodynamics (PKPD) model for metronomic chemotherapy using gemcitabine, one that considers the effects of cytotoxicity on endothelial cells and the emergence of drug resistance. Ledzewicz et al. proposed a minimally parameterized mathematical model for low-dose metronomic chemotherapy that explicitly considers tumor vasculature (66), and in subsequent work (67) applied optimal control theory to this system, so as to devise a treatment schedule that can minimize tumor burden subject to appropriate constraints. To the best of our knowledge, only one study has looked at modeling the immune recruitment by ICD drugs (62). In that work, however, there was no experimental validation of the proposed model.

Numerous cancer models have been proposed to account for the emergence of therapeutic resistance due to cancer cell heterogeneity. See, for example, the extensive references in Greene et al. (68). However, to our knowledge, no previous work has systematically and theoretically modeled what we call “systemic drug resistance,” by which we mean resistance as an immune-mediated dynamical phenomenon.

Here, we propose a mathematical model that is fit to experimentally observed tumor growth curves in GL261 tumor-bearing SCID mice that were given metronomic chemotherapy at Q6D to Q12D drug administration regimens (12). The proposed model incorporates immune cell recruitment, as well as pharmacokinetics of cyclophosphamide. First, we find a fixed set of model parameters, not adjusted for individuals or for drug schedule, that fit experimental data across these various drug regimens very well. To further validate the model, we not only compare the experimental fits to the measured tumor volume data, but also ask if the “latent variables” in our model, which represent immune activation, can reproduce a second set of experimental data, not used in training the model. Specifically, we asked if peak immune activation times in our model correspond to experimentally measured times. Finally, we investigate how our validated model can be used as a tool to identify better treatment schedules, to build a quantitative understanding of the mutual interplay between drug, tumor, and immune system and to better predict drugs that induce immune cell recruitment in a clinical setting.

## 2. Materials and Methods

### 2.1. Background on Cyclophosphamide and Experimental Results

#### 2.1.1. Summary of Experimental Data

The experimental data used in developing our mathematical model were derived from previous work by Wu and Waxman (12), where cultured GL261 gliomas cells were implanted in SCID mice. Tumors were allowed to grow to 300–1,000 mm^3^, at which point the mice were treated with cyclophosphamide (CPA), given on different metronomic schedules. Greatest tumor burden reduction was observed when repeated doses of 140 mg CPA/kg-BW (body weight) were administered every 6 days. Comparisons were made between the every 6-day schedule (Q6D) and three other schedules: treatment every 9 days (Q9D); treatment alternating between every 6 days and every 9 days; and treatment every 12 days (Q12D). Additionally, a dose of 210 mg CPA/kg-BW was given every 9 days, ensuring the mice receive the same total amount of drug as when 140 mg/kg doses were given on a Q6D schedule, to evaluate the impact of schedule vs. total dose on the final outcome. Tumor growth curves were reported for drug-free controls, and for the following regimens: a single CPA administration given on day 0 (1-CPA), two CPA treatments given on days 0 and 6 (2-CPA), as well as three CPA treatments given on days 0, 6, and 12 (3-CPA). This previous work also reported relative gene expression levels for immune cell markers for NK cells, dendritic cells, and macrophages for the 1-CPA, 2-CPA, and 3-CPA treatment regimens. There were *n* = 4 − 12 tumors per treatment group.

Published data (12) suggest that the host immune system takes between 6 and 12 days to start significantly impacting the tumor growth. From this, the authors infer that any initial slowdown in tumor growth, within 1 or 2 days of drug injection, is likely caused by cyclophosphamide-induced tumor cell death. A decrease in immune cell number immediately after drug administration was observed, highlighting the drug's cytotoxic effect on immune cells as well as cancer cells. Notably, the chemoattractant CXCL10, which acts on many innate immune cells, and which is induced by IFN-λ, increased following the first CPA injection, peaking around 6 days post administration. Between 6 and 12 days post injection, there was also an increase in other innate immune cells markers, such as NKp46, NKg2d, Prf1, Gzmb for NK cells, CD207, and CD74 for dendritic cells, and CD68 and Emr1 for macrophages (12).

Based on the impact of various treatment schedules tested on tumor volume, it is apparent that 12–18 days after CPA treatment is halted, the GL261 tumors cease to shrink and then start to regrow. Based on treatments where the same dose of CPA is repeated at regular intervals, the authors concluded that a 6-day break between CPA doses is ideal for maintaining prolonged immune cell recruitment as well as tumor shrinkage. Increasing the number of drug-free days between treatments from 6 to 9 to 12 days increased the number of tumors that circumvented the therapeutic effects of the drug, resulting in a shorter interval prior to tumor regrowth (13). Further analysis of tumor infiltrating immune cells in these models indicated there is a strong correlation between loss of immune response and tumor escape. Another study indicated that breaks in drug treatment shorter than 6 days also resulted in worse performance for the same AUC (area under the curve for the administration of the drug) with noted absence in immune recruitment if CPA was given to the mice daily (13). One of the first goals of our model is to recapitulate these results.

### 2.2. Modeling the Immune-Tumor-Drug Interactions

**Figure 1:**
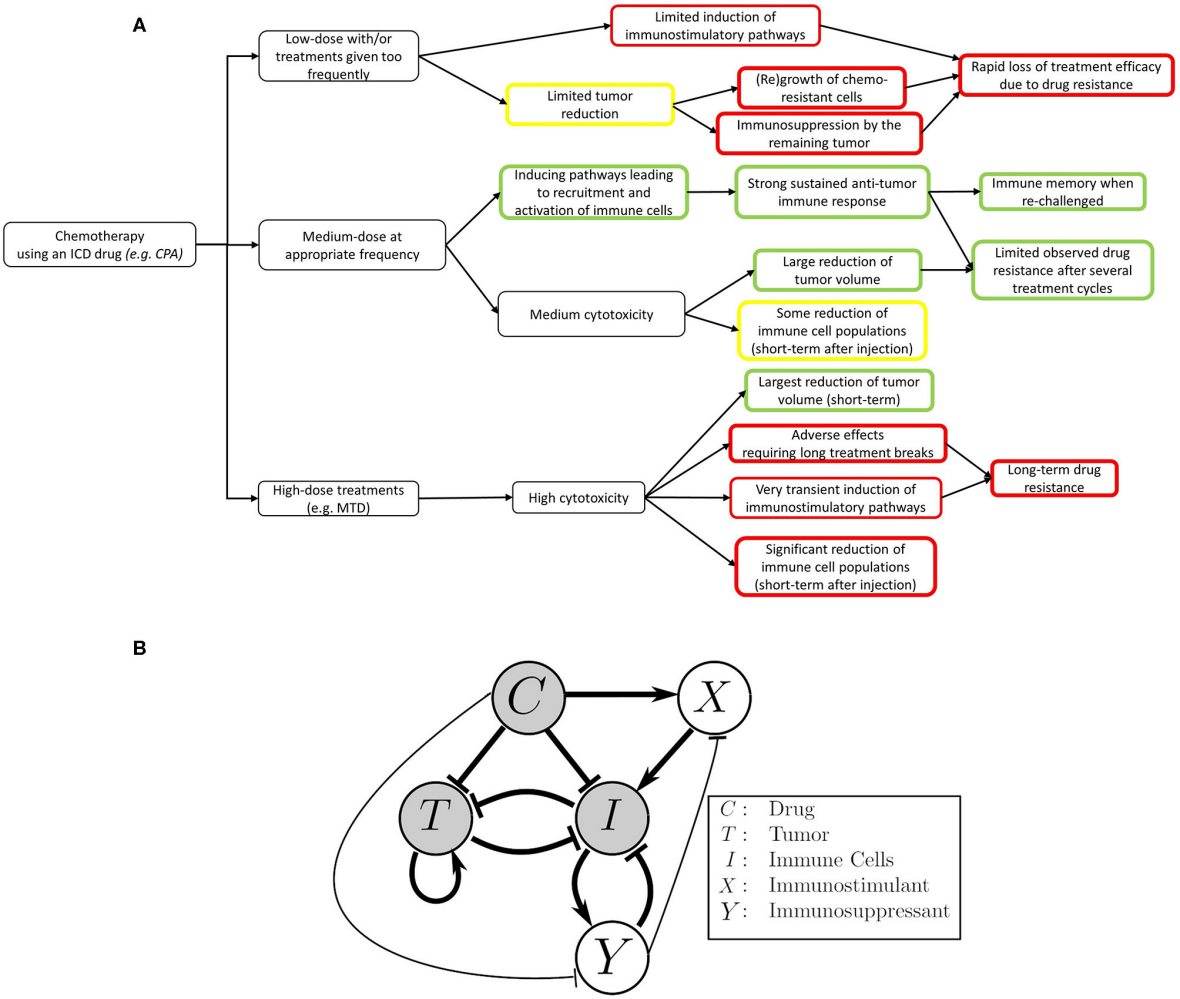
Mobilizing and sustaining a strong immune response requires striking the right balance between drug dosage and length (duration) of the drug-free break between drug administrations. The classical view of chemotherapy treatments is that the chemotherapeutic drug suppresses the immune system and is toxic to the tumor cells, while the tumor and immune system repress one another. In this view, the immune system's ability to act on cancer cells is greatly reduced by repeated cytotoxic drug administrations. The immune system can also be recruited to act on cancer cells by, for example, stimulating antigen presentation. The focal point of this work is to improve our mechanistic understanding on how application the right metronomic chemotherapy regimen can, in specific cases, stimulate a large increase in the immune response. Both the immunosuppressive and immunostimulatory effects of the drug are considered in our model, with the coexistence of these two seemingly opposite effects being an important point of emphasis. **(A)** An outline of the effects of different chemotherapy treatments on the immune system and tumor growth as observed in the context of an ICD drug like cyclophosphamide (17). **(B)** The translation of the outlined experimental observations into a detailed schematic diagram of the interactions that formed the basis of our model. Thicker arrows represent well-known effects. Thinner arrows are hypothesized to be present, and also arise from our numerical fits to the data. Notably, we simplified the system such that the interactions between tumor and immune cells leading to increased immune activity are omitted in the context of this work. The details about this assumption can be found in the Methods section.

A schematic of our model, highlighting the main interactions between tumor, drug, and the immune system, is shown in Figure 1A.

Cyclophosphamide can be toxic to the immune system, as confirmed by experimental data showing that, one day after cyclophosphamide administration, there is a significant decrease in marker gene expression for NK cells, DCs, and macrophages in the tumor microenvironment (12). High doses of cytotoxic drugs that damage immune cells can diminish or even nullify their ability to target the tumor. Given this finding, it is not surprising that traditional MTD chemotherapy not only has substantial side effects on the patient's health, but also leads to immunosuppression and increases the risk of tumor relapse due to drug resistance. This then naturally leads to the question of how to represent (in a concise but not oversimplified mathematical way) the immunostimulatory and immunosuppressive effects of drug treatment when using a metronomic regimen of an ICD drug in order to eventually find a way to balance out these two forces.

The paradoxical effect in which a drug reduces immune cell counts in the short term, but also enhances the immune system in the longer term, is an instance of an “incoherent feed-forward loop” (IFFL). A similar paradoxical effect is that of the effect of treatment on cancer growth: on the one hand, the drug directly attacks the tumor, but on the other hand, through “friendly fire” also attenuates immune activity, thus degrading the anti-tumor response. IFFLs constitute one of the core network motifs in systems biology (69), and are found in processes as varied as gene regulation, immune recognition, synthetic biology, biological sensors, and bacterial motion (70–78).

**Figure 2:**
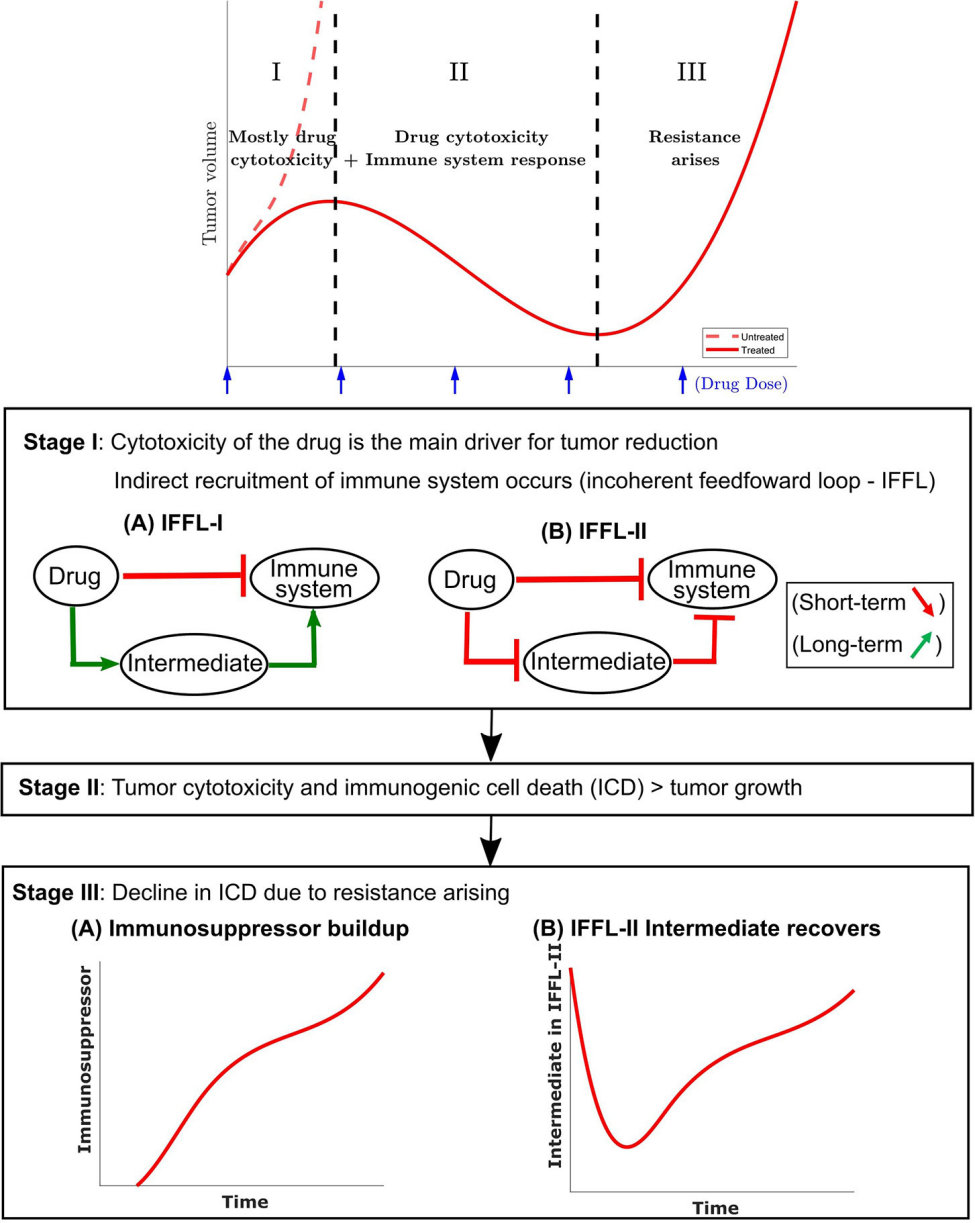
A conceptual tumor growth curve under metronomic chemotherapy treatment with repeated doses is shown and is broken down into three representative stages. In stage I, tumor growth is primarily inhibited by the direct tumor cell cytotoxicity of the drug. There is typically a delay in the immune response due to the immune cell cytotoxicity of the injected drug. The immune cell data in Wu and Waxman (12) indicate that an immune response is substantially reduced immediately after drug administration, but eventually recovers and leads to a strong response 6–12 days later. Two possible incoherent feed-forward loop (IFFL) motifs that capture these short-term and long-term dynamics of the drug-immune system interactions are shown. In stage II, the tumor volume is typically reduced as the induced immune response becomes the principal contributor to tumor cell death. Stage II lasts until the induced immune response fades due to the emergence of drug resistance. Stage III typically has the tumor recovering its ability to grow in an exponential fashion. Two possible scenarios are shown with the occurrence of an immunosuppressor build up or recovery of the intermediate in an IFFL-II scenario.

Some of the most common forms of IFFLs are illustrated by IFFL-I and IFFL-II block diagrams in Figure 2. In our context, two forms of IFFL, promotion of an immunostimulatory intermediate (IFFL-I) and “repressing the repressor” (IFFL-II), are both likely scenarios, and could occur in conjunction. Accordingly, to represent the immune system recruitment behavior, the mathematical model must include an intermediate that leads to immune cell recruitment in the longer term. The possible mechanisms are numerous, as summarized in section 1, and it is likely that the observed tumor growth is the result of both a repressor being repressed and some immunostimulatory element. Due to the lack of extensive immune data measurements, we only considered the immunostimulatory pathway in this work.

In the event of tumor escape depicted conceptually in the bottom section of Figure 2, the experimental data in Wu and Waxman (12) suggests that the immune system, which was reinforced through the first few injections of cyclophosphamide, fails to maintain a level of immune cell recruitment sufficient to allow the host to keep the tumor at bay.

### 2.3. Mathematical Model

The full mathematical model is given by a coupled system of five ordinary differential equations (ODEs). The first equation describes the change in CPA concentration over time (drug pharmacokinetics):

(1) $$\begin{array}{l} \frac{dC}{dt}=u-\frac{k_{1}C}{k_{2}+C} \end{array}$$

and the remaining four describe the effect of CPA on cancer and immune cells (pharmacodynamics):

(2) $$\begin{array}{l} \frac{dT}{dt}=k_{a}T-\frac{k_{b}CT}{k_{c}+T}-k_{d}TI \end{array}$$

(3) $$\begin{array}{l} \frac{dI}{dt}=X-k_{e}TI-k_{f}CI-k_{g}YI-k_{h}I \end{array}$$

(4) $$\begin{array}{l} \frac{dX}{dt}=\frac{C}{1+C/k_{i}}-k_{j}X-k_{k}XY \end{array}$$

(5) $$\begin{array}{l} \frac{dY}{dt}=\frac{I}{1+C/k_{l}}-k_{m}YC \end{array}$$

In the next section we describe in detail the various terms of the model, including the role of the variables *C* = *C*(*t*), …, *Y* = *Y*(*t*), which represent time-dependent changes of drug, tumor, and immune components, as well as provide descriptions and interpretation of the parameters *k*_1_, …*k*_*n*_. The input variable *u* = *u*(*t*) is introduced to describe drug administration. The equation terms with missing parameters are the result of a non-dimensionalization step that is described detail in Supplementary Note 1. It facilitates model analysis without loss of utility.

#### The PK Submodel

Equation (1) describes the change over time in concentration of cyclophosphamide *C*(*t*) in the tumor microenvironment. It is assumed that the drug is administered at a time-dependent rate *u*(*t*) and is cleared at a Michaelis-Menten (saturated) rate $\frac{k_{1}C}{k_{2}+C}$. This compartment is assumed to be where the interactions between the drug and its targets are assumed to take place.

Parameters *k*_1_ and *k*_2_ are obtained as part of the global fit to experimental data described later.

Our simple PK model is phenomenological and is intended to capture the delay in drug activity with respect to various cell types rather than all the details of CPA activity, which is sufficient for the purposes of our investigation. Furthermore, although the steps of CPA activation by liver cytochrome P450 metabolism have been well-studied, including the pharmacokinetics of CPA in mice (79, 80), details of how the intermediates interact in real time with the immune system and the tumor cells remain largely unknown. In using a one-compartment model, the fast dynamics of the drug reaching the blood stream and the decomposition of CPA into its metabolites are assumed to have occurred (as captured by the term *u*(*t*)) prior to the ability of the drug to impact system dynamics, reflecting the difference in time scales of drug PK and tumor-immune dynamics.

#### Tumor and Immune Dynamics

The full model describes interactions between five variables. These represent the tumor volume, denoted by *T*(*t*), the immune response denoted by *I*(*t*), and two phenomenological variables: an immunostimulatory intermediate species and an immunosuppressive intermediate species, whose counts are denoted by *X*(*t*) and *Y*(*t*), respectively. All these variables except *T*(*t*) are phenomenological representations of complex underlying phenomena, lumping together both cellular populations and chemical signals such as cytokines, and thus do not carry any biologically meaningful units. Such an approach allows identifying broad functional classes of actors that impact the observed dynamics, which can then be teased out in more detail in future investigations. All three of these species are directly affected by the concentration *C*(*t*) of cytotoxic drug.

We assume that tumor volume grows exponentially, at a rate *k*_*a*_. In contrast to other mathematical models, we do not introduce saturation or crowding terms for tumor growth, because the data used in fitting is from mouse models, where a maximum tumor volume (or carrying capacity) is never reached for humane reasons. In our model, tumor cells can be killed by two different mechanisms: either by interaction with the drug *C*(*t*) at a rate *k*_*b*_, or by immune cells *I*(*t*) at a rate *k*_*d*_. The ratio $\frac{T}{k_{c}+T}$ captures the fact that the cytotoxic death term is proportional to *T* when the tumor is small but saturates when *T* becomes much larger than *k*_*c*_.

Taking into account these mechanisms results in the following equation for change in tumor size over time:

(6) $$\begin{array}{l} \frac{dT}{dt}=\underset{\text{exponential growth}}{\underset{︸}{k_{a}T}}-\underset{\text{cytotoxic death}}{\underset{︸}{\frac{k_{b}TC}{k_{c}+T}}}-\\ \underset{\text{death as a result of immune-tumor interactions}}{\underset{︸}{k_{d}TI}}. \end{array}$$

For the purposes of this analysis, we do not differentiate between various types of cytotoxic immune cells nor do we distinguish the effects of these cells from other immune factors such as chemokines or cytokines; instead, we track the change over time of an aggregate immune indicator *I*(*t*). We assume that this indicator increases through direct interaction with immunostimulatory intermediate *X*(*t*) (which will be described next), and can either be inactivated through interactions with tumor cells at a rate *k*_*e*_, can decrease (“or die if seen as immune cells”) due to exposure to drug *C*(*t*) at a rate *k*_*f*_, can become suppressed through interaction with immune suppressor *Y*(*t*) at a rate *k*_*g*_, and can decrease at a natural rate *k*_*h*_. The term −*k*_*e*_*TI* represents both the activation *k*_*e*+_*TI* and inactivation of the immune response by tumor cells −*k*_*e*−_*TI* with the assumption that −*k*_*e*_*TI* = *k*_*e*+_*TI* − *k*_*e*−_*TI*. While fitting these parameters to data, we found that defining the tumor-immune interaction term in this particular functional form, i.e., −*k*_*e*_*TI*, led to a positive *k*_*e*_ value. This observation was likely due to the data being collected at a late stage in tumor growth progression. While one may expect the activation and inactivation phenomena to have different functional forms, the simplification was also made due to the experimental data suggesting that the recruitment by a *k*_*e*+_*TI* term on the immune cells, cytokines, and chemokines is smaller by at least an order of magnitude when compared to the immune recruitment driven by the metronomic chemotherapy treatment.

This results in simplification of the following equation for change in the indicator *I* over time from:

(7) $$\begin{array}{l} \frac{dI}{dt}=X-\left(k_{e-}-k_{e+}\right)TI-k_{f}CI-k_{g}YI-k_{h}I, \end{array}$$

to:

(8) $$\begin{array}{l} \;\frac{dI}{dt}=\underset{\text{drug-mediated immune recruitment}}{\underset{︸}{X}}-\underset{\text{exhaustion}}{\underset{︸}{k_{e}TI}}-\underset{\text{death by drug}}{\underset{︸}{k_{f}CI}}-\\ \underset{\text{immunosupression}}{\underset{︸}{k_{g}YI}}-\underset{\text{decay}}{\underset{︸}{k_{h}I}}. \end{array}$$

Next, in addition to tracking the dynamics of tumor and immune cells, we introduce two phenomenological variables that are both affected by the drug, and can in turn affect both tumor and immune cells.

Firstly, we introduce an immunostimulatory intermediate *X*(*t*), which impacts immune cell recruitment. We assume that it can be increased by an ICD type drug, such as cyclophosphamide, according to saturating function $\frac{C}{1+C/k_{i}}$, where immune cell recruitment is linear up to a threshold *k*_*k*_, and becomes saturated when *C*(*t*) > *k*_*i*_, representing an upper bound of drug-induced immune recruitment. The immunostimulatory intermediate *X*(*t*) is assumed to decay at a rate *k*_*j*_, and to be inactivated through interactions with an immunosuppressive factor *Y*(*t*), which will be defined next. The resulting equation for change over time of immunostimulatory factor *X*(*t*) is as follows:

(9) $$\begin{array}{l} \;\frac{dX}{dt}=\underset{\text{recruitment by drug}}{\underset{︸}{\frac{C}{1+C/k_{i}}}}-\underset{\text{decay}}{\underset{︸}{k_{j}X}}-\underset{\text{immunosupression}}{\underset{︸}{k_{k}XY}}. \end{array}$$

Finally, we introduce the immunosuppressive factor *Y*(*t*), which can impact tumor-immune dynamics and which in itself is affected by the drug *C*(*t*). Its dynamics over time are described by the following equation:

(10) $$\begin{array}{l} \frac{dY}{dt}=\frac{I}{1+C/k_{l}}-k_{m}YC. \end{array}$$

The immunosuppressive intermediate *Y*(*t*) is assumed to be induced by *I*; its effectiveness can be affected by drug *C*(*t*), which is captured as $\frac{1}{1+C/k_{l}}$. It can also be cleared through interaction with the drug *C*(*t*) at a rate *k*_*m*_.

The model accounts for cytotoxic effects of cyclophosphamide, not only on cytotoxic immune cells, such as NK and CD8^+^ T cells, but also on immunosuppressive cells, such as MDSCs and Tregs (61, 81, 82). The immunosuppressive intermediate *Y* is vital to the appearance of systemic drug resistance when the treatments are continuously repeated over long treatment periods.

A schematic summary of these interactions is shown in Figure 1B. A structural identifiability analysis was performed using (83) and showed that all parameters are (locally) identifiable.

## 3. Results

In this section, we first use the phenomenological model described above to fit experimentally observed tumor growth curves. We then validate the model by comparing model predictions for the immune compartment to experimental data; notably, the data for the immune compartment were not fit, and thus provide an independent validation of the model, where a single set of parameter values was sufficient to recapitulate experimentally observed dynamics. Finally, the validated model is used to make predictions about treatment regimens that have not been tested experimentally.

Here, the notations 1-CPA, 2-CPA, 3-CPA are used to indicate 1, 2, or 3 doses of CPA that are given 6 days apart. The first dose is always given on day 0. CPA/6-days, CPA/9-days, CPA/12-days indicate that treatments were given 6, 9, or 12 days apart, respectively. CPA/9-days(210 mg/kg) indicates that the drug doses of 210 mg/kg (rather than 140 mg/kg in other treatment groups) were administered 9 days apart. Finally, the abbreviation CPA/6-9-days indicates a break of 6 days between first and second doses, and a break of 9 days between second and third doses.

### Population Fits to the Experimental Data

The experimental growth curves for individual tumors were obtained from Wu and Waxman (12) and fitted using the objective function outlined in Supplementary Note 2. The minimization of the error criterion was carried out using *fmincon* with the interior-point algorithm in MATLAB (Release R2019a, Mathworks, MA).

The initial values of the state variables were assumed to be 0 except for tumor volume. The rationale behind this assumption is detailed in Supplementary Note 2. The initial guesses to the optimization problem used for finding population parameter fits were drawn from a uniform distribution on a log-scale to sample several orders of magnitude. Numerous initial guesses (>10^5^) were tested using the Northeastern Discovery computer cluster. Due to the nonlinearity of the model, multiple sets of parameters yielded equally good minimal objective function evaluations. Out of 16,000 starting points for the optimization, the 8 fits within 1% of the objective function optimal value are summarized in Supplementary Table 1. Outliers highlighted in Supplementary Figure 2 were excluded using the procedure outlined in Supplementary Note 3.

**Figure 3:**
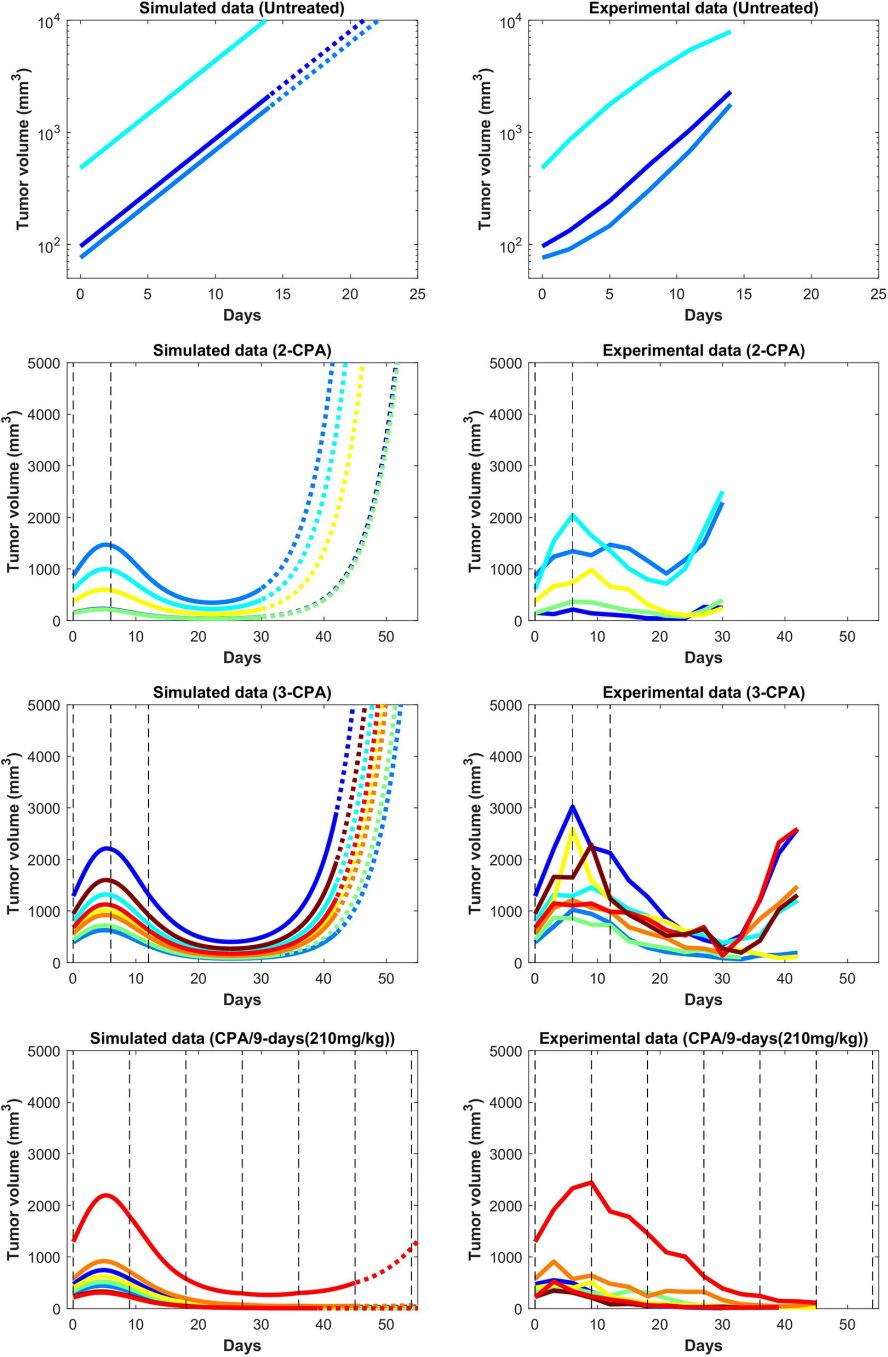
Simulated and experimental growth curves for the scenarios of 1-CPA, 2-CPA, 3-CPA, and doses of 210 mg/kg given every 9 days (CPA/9-days/210). When not specified, CPA doses are 140 mg/kg. Black dashed lines represent the time at which drug treatments are given. Solid lines represent fitted data; dotted lines represent predicted growth curves extrapolated from the model.

**Figure 4:**
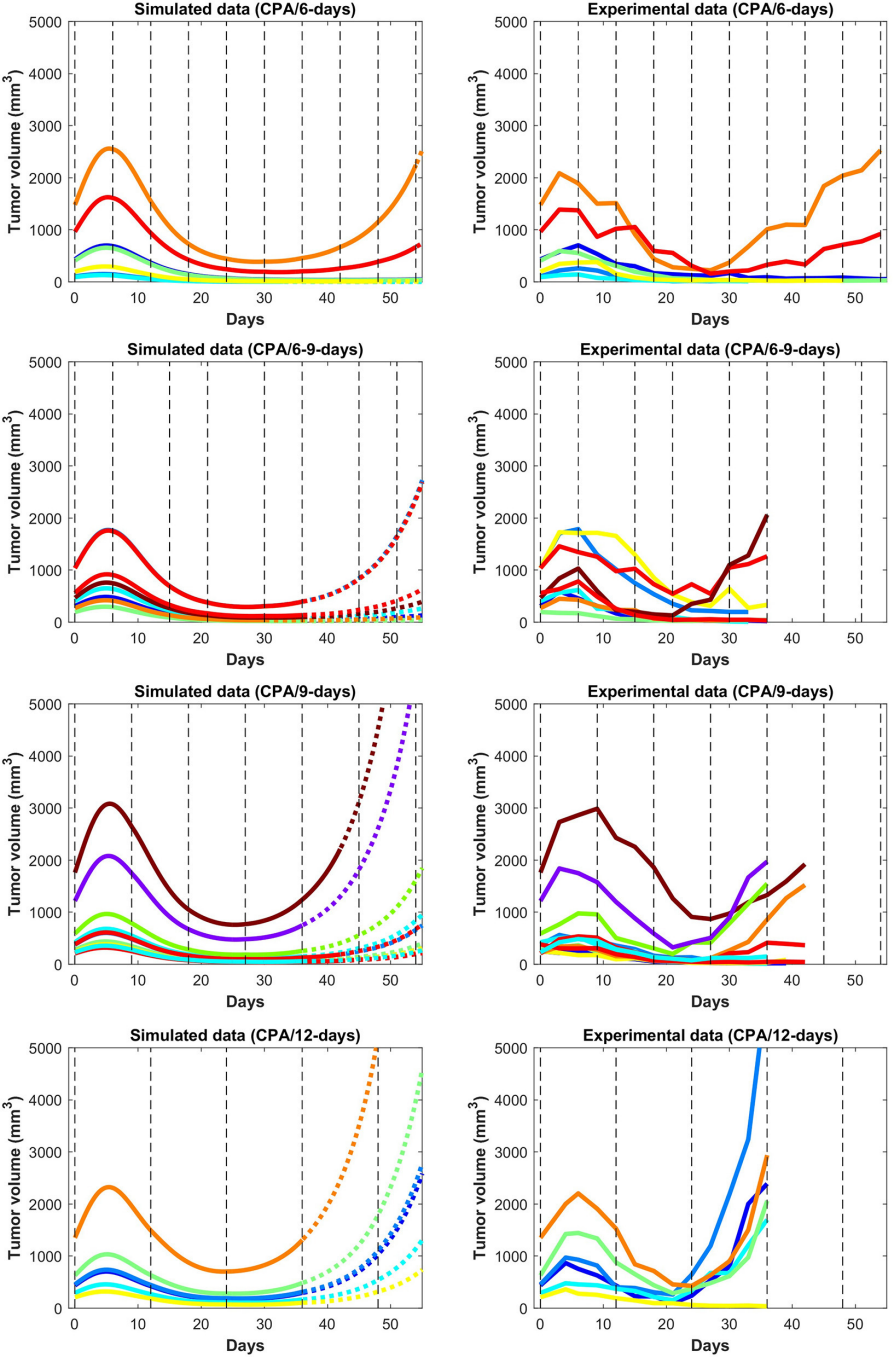
Simulated and experimental growth curves for the scenarios CPA/6-days, CPA/6-9-days, CPA/9-days, and CPA/12-days. Solid lines represent fitted data; dotted lines represent predicted growth curves extrapolated from the model.

In Figures 3 and 4, the simulated growth curves generated using fitted parameters presented as in Fit A in Supplementary Table 1 are shown side by side with the corresponding experimental data. This Fit A was picked among the 8 best fits due to it better capturing the qualitative behavior for CPA/12-days, but the differences between these fits among the other sets of treatment conditions were relatively small. Additionally, the predicted pharmacokinetics are shown in Supplementary Figure 3. It is important to note that each treatment condition corresponds to an experimental dataset. Consequently, variations between datasets can be more prominent than within the same dataset. Also, the units were not emphasized due to three out of the five state variables being dimensionless. However, it is straightforward to adapt the proposed model in the presence of such experimental data by applying the non-dimensionalization transformations outlined in Supplementary Note 1 and obtain the appropriate units.

Despite the use of pooled mouse data for curve fitting, there is a high degree of agreement between experiments and model fits, particularly for tumors that remained largely suppressed throughout treatment. The nature of rebounding (or escaping) tumors makes the observations very stochastic in nature, while the models are progressively transitioning between regressing and rebounding tumors under conditions as the initial tumor volume gets larger (an example of this transition being the CPA/6-9-days treatment). Allowing for small variations in the parameters between individual experiments is likely to account for such variability. The primary intent is to showcase that one unique set of population parameters can capture the qualitative and quantitative behaviors of a large set of different metronomic chemotherapy treatments that involves induction of anti-tumor immune responses and drug resistance.

The agreement between simulated and fitted experimental growth curves is high for the untreated, 2-CPA, 3-CPA, and CPA/9-days(210 mg/kg) cases, as can be seen in Figure 3. There is a discrepancy between the model prediction and the experimental data for the largest tumor in the 2-CPA scenario. In the repeated treatments, the model captures well the progressive apparition of rebounding tumors as intervals between drug administration increase from 6 to 12 days in 4 increments. For the CPA/12-days scenario, the model predicts that tumor escape will occur up to ten days after when it actually occurred experimentally; nevertheless, the model is able to capture the qualitative effect of this treatment regimen, which fails rapidly within the first 3 drug injections.

The 1-CPA set of data was small in size (*n* = 5, before an outlier was excluded) and tumors that were implanted apparently grew at a significantly faster rate than the rest of the dataset. Given that each metronomic scenario was from a different batch of mice, systemic experimental variations in the fitted data can account for some of these observed discrepancies and can be hard to distinguish from model deficiencies. From Supplementary Figure 1, the three treatment conditions of 1-CPA, CPA/9-days, and CPA/12-days are not expected to differ until day 9, but the plots indicate that the experimental data is inconsistent. In addition, 1-CPA and CPA/12-days have mice undergoing the same treatment conditions until day 12.

**Figure 5:**
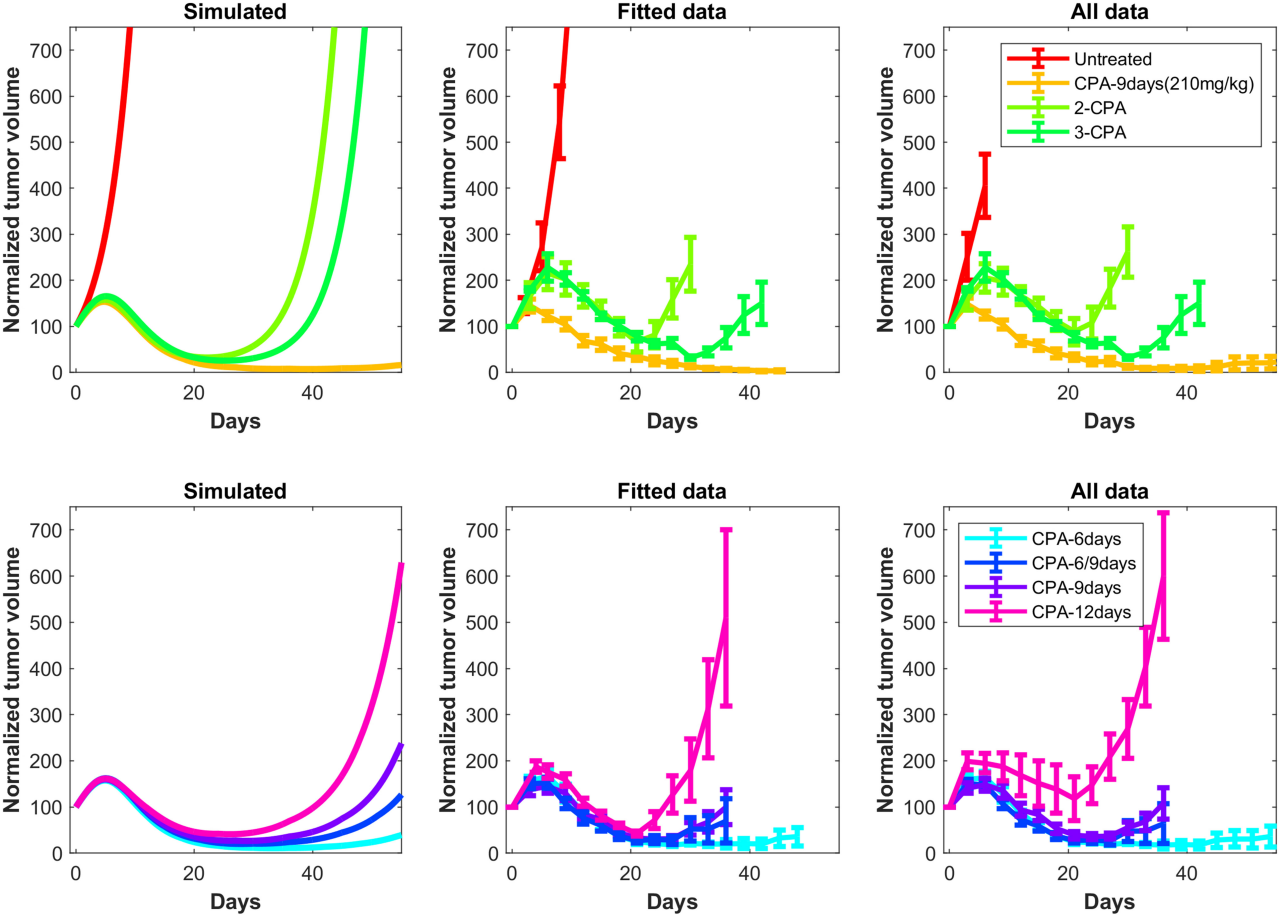
Simulated tumor growth curves of all the considered treatments scenarios were normalized such that the first time point of each curve has an initial value of 100. The mean of the normalized curves for each treatment condition was then calculated and plotted on the left panels. The right panels are the original experimental data from Wu and Waxman (12). These data also show the difference between the normalized average fits of the fitted data without outliers (middle panels) and the experimental data including outliers (right panels). The outliers are highlighted in Supplementary Note 3.

In Figure 5, the mean normalized tumor volumes are given for each of the conditions modeled and shown with the corresponding experimental data. There is a slight delay in the 2-CPA case when it comes to the rebounding behavior of the tumors. The CPA/12-days discrepancy in the time of rebound can also be seen. Notably, the deficiencies in the fits are more pronounced on the marginal cases of longer breaks and shorter treatments. It is possible that these edge cases require special attention to incorporate the impact of other phenomena that are discussed in Wu and Waxman (17), and might require additional experimental data to appropriately capture the underlying mechanisms. Also shown in Figure 5 is the difference between the normalized average simulations of the fitted data without outliers and the experimental data with outliers. The trends were not affected by removing outliers with the exception of CPA/12-days that yielded large discrepancies between 4 and 25 days. This was due to two small tumors that were excluded as they grew very rapidly to be much larger than other tumors that had higher initial volumes.

### Predictions Regarding the Immune System

**Figure 6:**
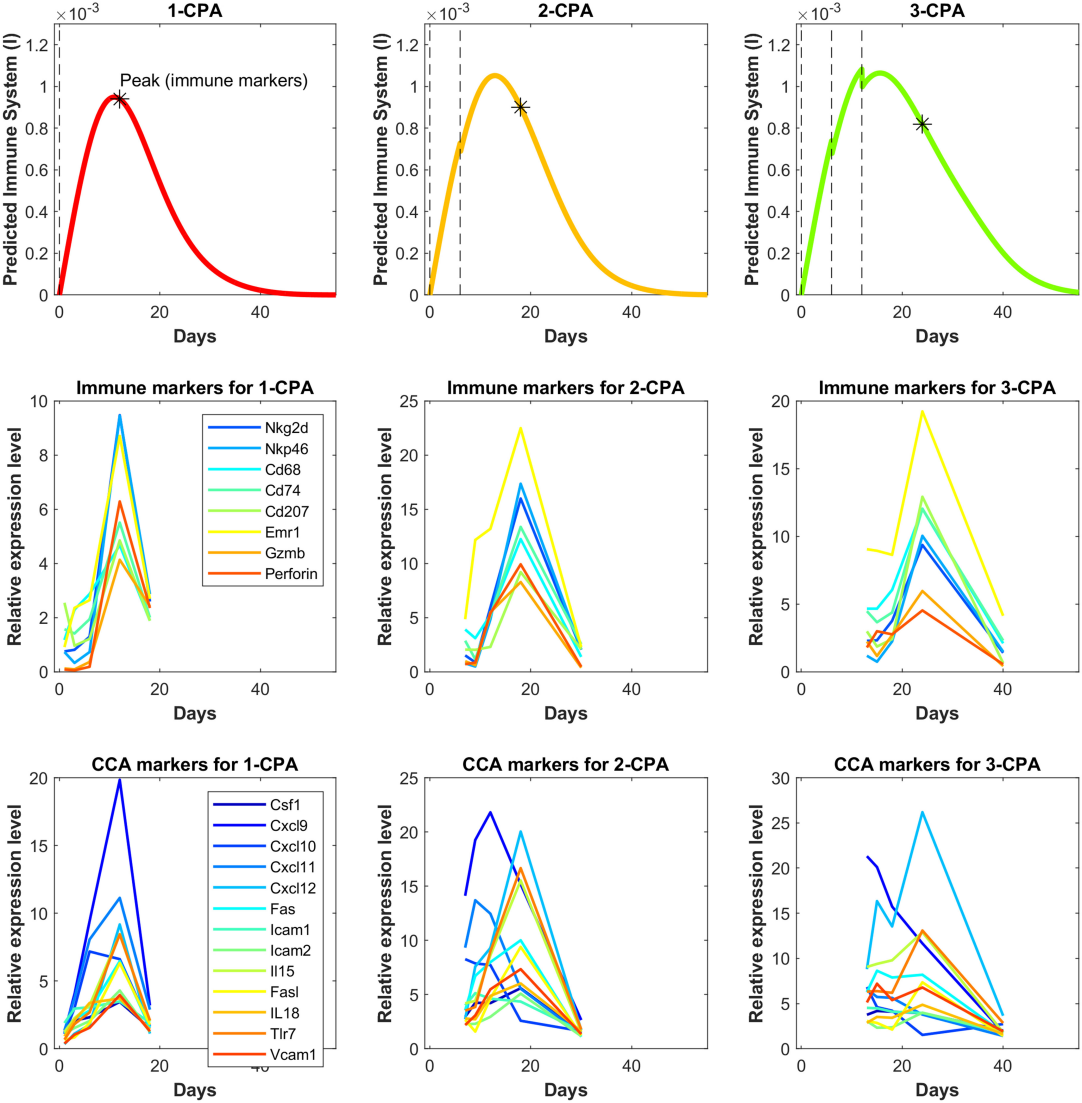
Independent model validation through comparison of model predictions to available immune cell data. The top row shows the predicted immune system recruitment by the fitted model for the 1-CPA, 2-CPA, and 3-CPA treatment conditions. The vertical black dashed lines indicate times of drug injections at 140 mg/kg. The initial volume for the simulated tumors yielding these curves was assumed to be 1,000 mm^3^ at the time of the first injection. The middle row shows the experimental data of the gene expressions for various immune markers linked to the innate immune cells (macrophages, dendritic, and NK cells). Similarly, the bottom row shows gene expression data for various chemokines, cytokines, and adhesion molecules (abbreviated CCA on the plots). The data is ordered such that each column represents the same treatment condition.

In Figure 6, predictions are made for 1-CPA, 2-CPA, 3-CPA for the immune system behavior. Notably, the immune data were never used in model fitting, but model predictions of the immune system correspond well to that of the immune cell data reported in Wu and Waxman (12). In this model, the immune system is assumed to be an aggregate of multiple immune cells and related factors. The experimental immune data, shown in Figure 6, can be separated into two categories: the immune cell markers and the chemokine, cytokine, and adhesion molecule markers. It is clear from the experimental data that new injections of the drug have a short term negative effect on immune cell populations. However, this is not often the case for cytokines or chemokines, which can be recruited and remain at high levels even after a second injection, as was the case for the 2-CPA regimen. Considering both effects of the immune cell markers and cytokines and related molecules, the predictions of immune system behavior by the model seem to be an aggregate of these responses. It estimates well the immune cell peaks that occur at 12, 18, and 24 days for 1-CPA, 2-CPA, and 3-CPA regimens, respectively. Notably, the experimental data are quite sparse, as only 4 time-points were analyzed for each treatment condition. Additional plots in Supplementary Figure 4 show the predicted immune system behavior for other treatment conditions.

### Predictions for CPA/9-6-days and CPA/7.5-days

**Figure 7:**
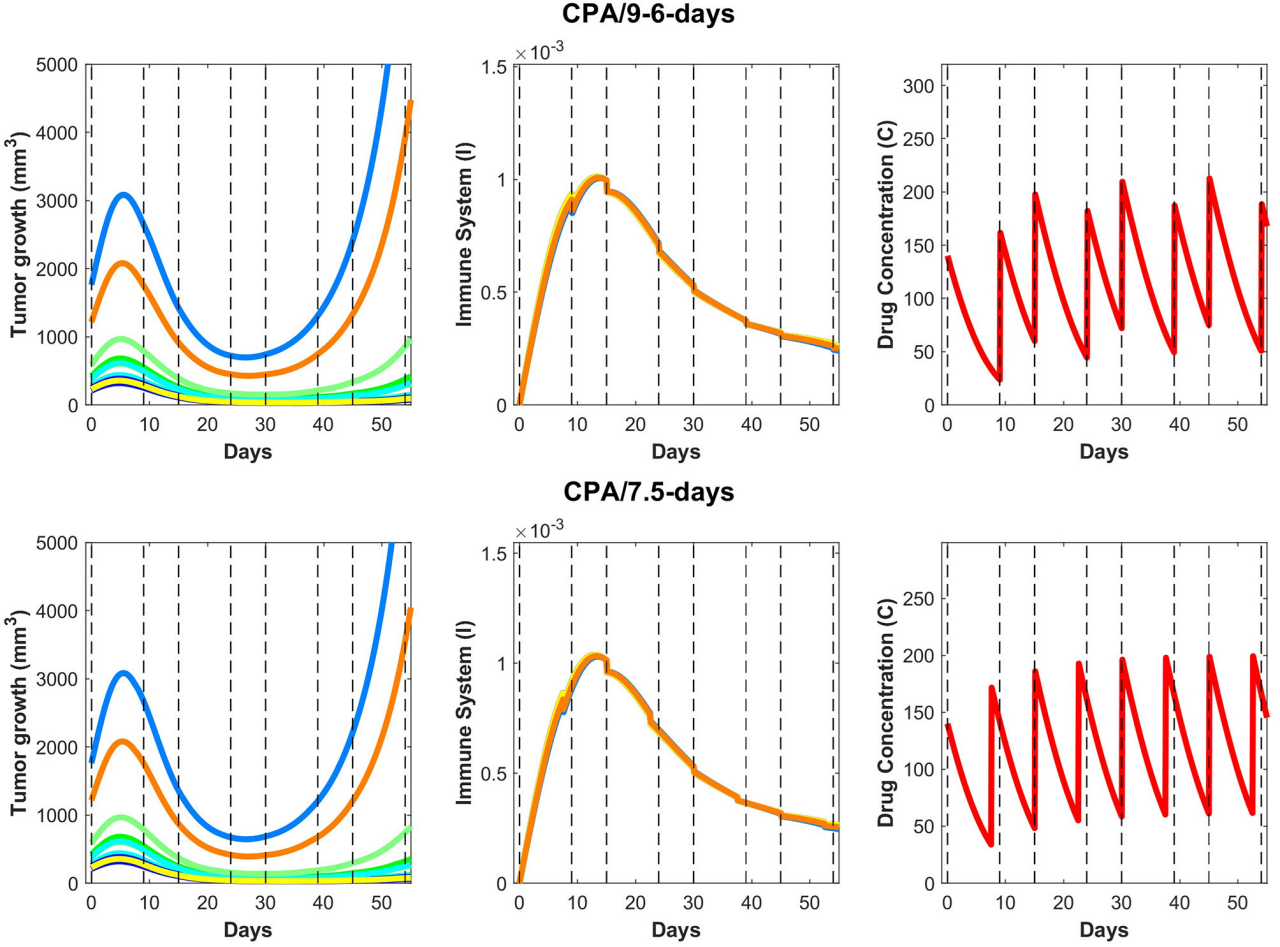
On the **(top)** row, predictions for the CPA/9-6-days drug regimen where the first break is 9 days followed by 6 days, then 9, then 6 and so on, alternatively. On the **(bottom)** row, similar predictions are made for CPA/7.5-days.

The validated model was then used to make predictions about the effect on tumor growth of dose administration regimens that were not experimentally tested. One such example is shown in Figure 7, where drug is administered at alternating breaks of 9 days and 6 days (CPA/9-6-days). Interestingly, the model predicts that CPA/9-6-days is considerably inferior to the CPA/6-9-days regimen, suggesting that shorter breaks between drug administrations early on improve outcomes, as compared to longer breaks. CPA/7.5-days is a little better than CPA/9-6-days, especially with smaller initial tumor volumes.

We hypothesize that shorter breaks early on allow sufficient tumor burden reduction to enable cytotoxic immunity to have greater impact on the smaller tumor. Notably, within this framework, the standard approach of maximizing tumor burden reduction would cause excessive damage to the immune system, so chemotherapy-induced tumor burden reduction should be sufficient to augment the effect of the immune system but not to act to its detriment. Based on our analysis, although the CPA/9-6-days schedule (Figure 7) should be superior to the CPA/9-days schedule (Figure 4), the two simulated datasets are almost identical in behavior.

Upon closer examination of relative impact in cancer cell death due to activity of the immune system vs due to the drug, the model indicates that even after the anti-tumor immune recruitment decreases, small tumors remain under control as a result of drug cytotoxicity. However, for large tumors, even a slight decrease in anti-tumor immunity can determine the difference between tumor regression and tumor progression. Looking at the immune response in Figure 7, a slight decrease around 15–20 days after the first treatment leads to strong rebounding behaviors in the two largest simulated tumors.

## 4. Discussion

The administration of cyclophosphamide under MTD regimens can be severely toxic to the immune system and undermine its ability to help control tumor growth. Changing the dose and timing of drug administration has been shown to restore the ability of cytotoxic immune cells to target tumor growth in glioma mouse models (12, 13), suggesting the existence of a “sweet spot” that can minimize damage to the immune system and maximize anti-tumor immune effects. To formalize the mechanisms that may underlie experimentally observed variation in response with respect to drug dose and schedule, we propose a phenomenological mathematical model that captures key processes that may underlie interactions between drug, immune system, and tumor. Through these efforts, we aim to identify key mechanisms that may give rise to a strong and sustained immune response, within the broader context of improving the understanding of cancer treatments that target not only cancer cells themselves but also the tumor microenvironment, and in particular, the immune system.

The proposed phenomenological model was fit using the method outlined in Supplementary Note 2 to generate a single set of parameters that was able to capture well tumor growth dynamics across nearly all experimentally tested drug treatments (Figures 3 and 4). The model was further validated through predicting the dynamics of the immune cells that were not used in the fitting process. We found a strong correspondence between immune data and predictions, despite the simplified nature of the underlying model (Figure 6). The parameter values of various fits within 1% of the optimal value for the objective function are plotted in Supplementary Figure 5. Notably, the tumor growth constant *k*_*a*_ was consistent among all the fits. This parameter is likely to be well-determined due to the abundance of tumor volume data and, in particular, the use of untreated tumor growth data in the fits. The parameters related to the tumor equation and the PK part of the model are generally within one order of magnitude among all the fits. The remaining parameters are mostly spanning several orders of magnitude and it is likely that a better correspondence of phenomenological variables *X*, *Y*, and *I* with the experimental data would alleviate this issue, namely that several combinations of parameters can redundantly capture the qualitative behaviors seen in the experimental data.

We then used the validated model to predict the impact on tumor growth of an alternative schedule of CPA/9-6-days as well as CPA/7.5-days that have not been tested experimentally (Figure 7). The model predicts that shorter breaks between dose administrations early on lead to greater tumor burden reduction and improved anti-tumor immunity; however, as was shown in Wu and Waxman (12) and Chen et al. (13), the breaks cannot be too short, which in turn may lead to excessive immune cell depletion, not allowing cytotoxic lymphocytes time for replenishment. Therefore, the goal of “sweet spot” therapy is to reduce tumor burden sufficiently to allow cytotoxic immunity to persist and control the tumor.

Despite the undeniable complexity of the immune system, the proposed conceptual model of four differential equations coupled with a 1-dimensional PK model allowed us to capture tumor responses to various treatment schedules. While the model was used to understand and reproduce data that shows impact of variation in dosing and scheduling on tumor growth for a particular mouse tumor model, it also highlights the fact that beyond simply understanding the interactions between the different components at play, a quantitative modeling approach may be able to help design better metronomic chemotherapy treatments. Furthermore, the conceptual nature of the proposed model enables us to pinpoint more specific mechanisms that are responsible for observed variations, which may not be possible with more detailed descriptive models.

Besides fitting well the experimental data on cyclophosphamide, the mathematical model developed here can be a valuable complementary tool to understand how drugs function and how they can be combined with other types of treatments, such as immunotherapies. It is also possible that drugs previously discarded due to a lack of cytotoxicity may have immunogenic properties that could act as effective complements to other treatments, a response that may have gone unnoticed in the context of MTD administration. Furthermore, the proposed framework suggests that what may appear as therapeutic resistance to a drug may in fact represent desensitization, a phenomenon that can be mitigated through alterations of the drug dosage and scheduling and through better understanding of how these different components of the tumor microenvironment interact with one another, particularly immune cells and cytokines.

One of the exciting properties of this model is that it demonstrates that the exhaustive details of specific immune cell and cytokine types are not necessary to explain experimentally observed data with regards to tumor growth; instead, we showed that grouping actors by function into “classes of cells” is surprisingly sufficient. Therefore, while it is tempting to try to introduce more details into equations describing either the immune cells or immunostimulatory and immunosuppressive factors, it may not provide additional insights. This is because the ultimate goal of this approach is not to quantify the immune system extensively, but to reliably predict long term tumor dynamics under treatment. Instead, it may be more beneficial to explore the impact of drug-specific kinetics on these different components. This approach is quite feasible, since pharmacokinetic modeling that describes experimentally measurable changes in drug concentrations over time has very well-developed methodology that is used extensively in drug discovery and development.

Drug-specific PK models can be coupled with the four Equations (2–5) to help better understand the push and pull of drug effects on all system components to get the ultimate measurable outcome: drug impact on tumor growth. Parameters that would need to be experimentally determined are those associated with drug-dependent toxicity on immune cells and cancer cells separately (within the frameworks of this model, they are parameters *k*_*b*_, *k*_*f*_, *k*_*m*_, *k*_*i*_, and *k*_*l*_), which can be measured in targeted experiments and then used as starting points for parameter optimization. The PK-PD model will then need to be trained on experimental data, as in the approach described here, and then used to evaluate the impact of different treatment regimens on final tumor growth. Notably, PK models can be created to describe the dynamics of both novel drugs and existing ones. This may open avenues to re-purposing existing drugs by appropriately altering the dosage and schedule of administration, an undertaking where quantitative approaches such as the one proposed here may prove to be indispensable.

The effectiveness of metronomic CPA schedules examined here has been demonstrated in syngeneic mouse glioma models, as well as in innate immune-sufficient SCID mice in work that includes mouse, rat and human xenografts; however, we do not know how predictive these models are of responsiveness in gliomas or in other tumor types in human patients. Evaluation of these metronomic CPA schedules in human tumor models, including patient-derived xenografts, is therefore a high priority for clinical translation.

We anticipate the proposed mathematical model will be useful for discovery of hidden potential of current drug treatments by building better quantitative understanding of the phenomena at play, and for designing more effective drug regimens that may not be intuitively apparent through exploring immunogenic potential of ICD drugs. The proposed model can also be used to investigate a variety of drug schedules and dosing regimens, as well serve as a building block for investigation of combination chemotherapy and immunotherapy treatments that will likely pave the future of cancer therapy.

## Data Availability Statement

The datasets generated for this study are available on request to the corresponding author.

## Ethics Statement

Ethical review and approval was not required for the animal study because the data used in this manuscript was already published previously.

## Author Contributions

IK and ES: conception and design. IK, AT, MA, and ES: development of methodology. JW and DW: acquisition of data. ES: administrative, technical, or material support. MA and ES: study supervision. All authors analysis and interpretation of data, writing, review, and/or revision of manuscript.

## Conflict of Interest

The authors declare that the research was conducted in the absence of any commercial or financial relationships that could be construed as a potential conflict of interest. IK is an employee of EMD Serono, a US subsidiary of Merck KGaA. Views expressed in this paper do not necessarily represent the views of EMD Serono.
