# Supplementary material for: Delicate Balances in Cancer Chemotherapy: Modeling Immune Recruitment and Emergence of Systemic Drug Resistance
Source: Front Immunol. 2020 Jun 30;11:1376. doi: 10.3389/fimmu.2020.01376 (PMC7338613; doi:10.3389/fimmu.2020.01376)
Supplement: Supplementary file 1 [file Data_Sheet_1.PDF]

Supplementary Information

Supplementary Note 1: Nondimensionalization of the model equations

Consider the equations of the model:

$$\frac{dC}{dt} = u - \frac{k_1 C}{k_2 + C} \tag{1}$$

$$\frac{dT}{dt} = k_{a'} T - \frac{k_{b'} C T}{k_{c'} + T} - k_{d'} T I \tag{2}$$

$$\frac{dI}{dt} = k_{e'} X - k_{f'} T I - k_{g'} C I - k_{h'} I Y - k_{i'} I \tag{3}$$

$$\frac{dX}{dt} = \frac{k_{j'} C}{1 + C/k_{k'}} - k_{l'} X - k_{m'} X Y \tag{4}$$

$$\frac{dY}{dt} = \frac{k_{n'} I}{1 + C/k_{o'}} - k_{p'} Y C \tag{5}$$

$$\tag{6}$$

Substituting I, X, and Y using the relationships:

$$\left[I = I^* \hat{I}, X = X^* \hat{X}, Y = Y^* \hat{Y}\right]$$

and limiting the scope to the equations for these state variables to be nondimensionalized yield the following set of equations:

$$\frac{d\hat{I}}{dt} = \frac{k_{e'} X_1^* \hat{X}_1}{I^*} - k_{f'} T \hat{I} - k_{g'} C \hat{I} - k_{h'} \hat{I} Y^* \hat{Y} - k_{i'} \hat{I} \tag{7}$$

$$\frac{d\hat{X}}{dt} = \frac{k_{j'} C}{X^* \left(1 + C/k_{k'}\right)} - k_{l'} \hat{X} - k_{m'} \hat{X} Y^* \hat{Y} \tag{8}$$

$$\frac{d\hat{Y}}{dt} = \frac{k_{n'} I^* \hat{I}}{Y^* \left(1 + C/k_{o'}\right)} - k_{p'} \hat{Y} C. \tag{9}$$

Making the following replacements

$$\left[I^* = k_{e'} k_{j'}, Y^* = k_{e'} k_{j'} k_{n'}, X^* = k_{j'}\right]$$

and rewriting the parameter names yield the following nondimensionalized set of equations with 4 less parameters:

$$\frac{dC}{dt} = u - \frac{k_1 C}{k_2 + C} \tag{10}$$

$$\frac{dT}{dt} = k_a T - \frac{k_b C T}{k_c + T} - k_d T I \tag{11}$$

$$\frac{dI}{dt} = X - k_e T I - k_f C I - k_g I Y - k_h I \tag{12}$$

$$\frac{dX}{dt} = \frac{C}{1 + C/k_i} - k_j X - k_k X Y \tag{13}$$

$$\frac{dY}{dt} = \frac{I}{1 + C/k_l} - k_m Y C. \tag{14}$$

Supplementary Note 2: Fitting methodology

Error criterion

In this work, an objective function was defined for the nonlinear optimization problem that is used to fit the model parameters:

$$\underset{\hat{y_i}}{\operatorname{argmin}} \sum_{i=1}^{N} \operatorname{sat}\left[\left(\frac{y_i + a}{y_i + b}\right)\left(\frac{|y_i - \hat{y_i}|}{y_i + c}\right)\right] \tag{15}$$

with y_i being the experimental value and $\hat{y_i}$ the predicted value. $\operatorname{sat}(x)$ is a saturation function such that $\operatorname{sat}(x) = 1$ for values of $x > 1$. The values of a, b, and c are determined in such a way that low values of y_i are not weighted too strongly. In the objective function, b and c can be interchangeable, so we will assume that $b < c$. In the limit that $b < y_i \ll a, c$, we get that:

$$\underset{\hat{y_i}}{\operatorname{argmin}} \sum_{i=1}^{N} \operatorname{sat}\left(\frac{a}{c}\frac{|y_i - \hat{y_i}|}{y_i + b}\right) \tag{16}$$

a and c are chosen such that $\frac{a}{c} < 1$ and b is small value that plays both the role of a regularizing term and avoids a division by 0. In the limit when y_i is large, we get:

$$\underset{\hat{y_i}}{\operatorname{argmin}} \sum_{i=1}^{N} \operatorname{sat}\left(\frac{|y_i - \hat{y_i}|}{y_i}\right) \tag{17}$$

which is a standard normalization. The magnitudes of a and c play an important role in how quickly this limit of large y_i is approached. Let's note that if $y_i \ll b \ll a, c$ then the ratio becomes $\frac{a}{c}\frac{|y_i - \hat{y_i}|}{b}$. The parameters are chosen such that $bc \gg a$, small values are filtered out, as these may be more susceptible to measurement noise or below a threshold of detection.

The values of a, b, and c in the error criterion were chosen as 19.31, 1, and 227.6, respectively. These values were found to provide a balance between providing enough weights to errors involving small experimental values, while also ensuring that the desired phenomena of tumor evasion and immune recruitment are captured appropriately by the model.

Determination of the population fits

The main underlying assumption of the population fits is that all mice are characterized by the same parameters. In reality, there can be a myriad of ways mice can differ from one another. Notably, their immune system might be of different strength when fighting the tumor and different tumors can grow at different speeds. However, the immune data that was available [1] is confined to the 1-CPA, 2-CPA, and 3-CPA scenarios and only three individual mice per experiment. The tumor growth curves were thus the only data that was used to fit the model.

In addition to having the same parameters, the initial values of all state variables were assumed to be 0, except for the initial tumor volume. While the mice do have a still functioning immune system at the moment they are being monitored, an escaping tumor is a sign that the immune system is compromised or unable to contain the tumor growth. Thus, the underlying assumption is that the immune system at the start of treatment is either of negligible effect or its effect on the tumor growth is lumped inside the k_a constant. Furthermore, the immune data from [1], also shown in Fig. 7, shows that the effect of cyclophosphamide given on a metronomic regimen can yield an order of magnitude increase in the gene

expression of innate cell immune markers, and similar observations can be seen for the gene expression of other markers for cytokines, chemokines, and adhesion molecules.

The last assumption for the population parameters is that the input u appearing in Eq. 1 consists of a step input of 140 mg/kg in concentration, applied at the moment a dose injection. From the time-scale of the experiments, the time-scale of the drug injection is negligible when monitoring the tumor size every few days. The input and the state variables besides the tumor (T) are assumed to be unitless. For the drug regimen of CPA/9-days(210mg/kg), the step input for u was increased to 210 mg/kg in concentration for each dose injection in order to account for the higher dose.

Supplementary Note 3: Handling the outliers

In this data, there are two types of outliers that were taken out of the analysis:

- Time series with average tumor volume above a given cutoff value of 2500 mm^3 and that did not belong to the untreated group.
- Time series that contradicted the behavior of neighboring curves.

The first criterion led to the exclusion of 3 outliers out of 65 time series. Large tumors followed dynamics different from what the model could explain. However, the large tumor data is very sparse, so there did not seem to be enough data to confidently elucidate the functional form that governs these data points.

Given the interest in finding a set of population parameters that captured the quantitative and qualitative behaviors of the experimental data, a second criterion was used to avoid fitting the model with contradictory behaviors. To quantitatively measure these deviations, the experimental values of the tumor volumes at each time point at a given treatment condition are ranked. Defining r_{ijk} that represents the rank for the time point i of time series experiment j at the treatment condition k. This rank can be used to form a rank vector R_{jk} such that:

$$R_{jk} = \{r_{1jk}, ..., r_{Njk}\} \text{ for } N \text{ time points.} \tag{18}$$

For each time series, a scalar D_{jk} is calculated using the following formula:

$$D_{jk} = \frac{\text{std}\,(R_{jk})}{M_k} \tag{19}$$

with std representing the calculation of a standard deviation and M_k the number of time series data at a given treatment condition k. When two or more tumors at a given time point and treatment condition had the same recorded tumor volume, the average rank of these tumors was assigned for r_{ijk}.

The outliers in the data are shown in Fig. 2 and were picked if they were singled out by the first criterion and/or second criterion. The exclusion rule for the second criterion was defined as $D_{jk} > 0.21$.

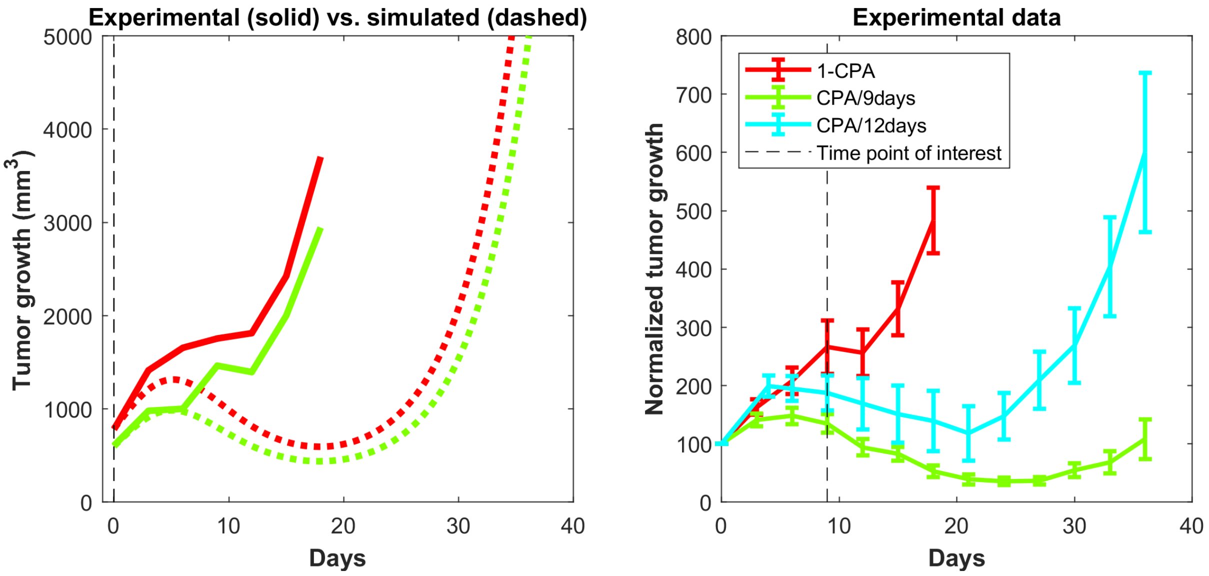

Supplementary Figure 1: Discrepancies in modeling 1-CPA. On the left, are shown the experimental data and the simulated data, with the latter data predicting much slower tumor growth than was seen in the experimental data. On the right, the 1-CPA data is plotted using the normalized tumor growth and compared with CPA/9-days and CPA/12-days treatments. Note that the 1-CPA treatment is equivalent to the CPA/9-days and CPA/12-days data up to treatment day 9 and treatment day 12, respectively, barring systemic errors in the experiments.

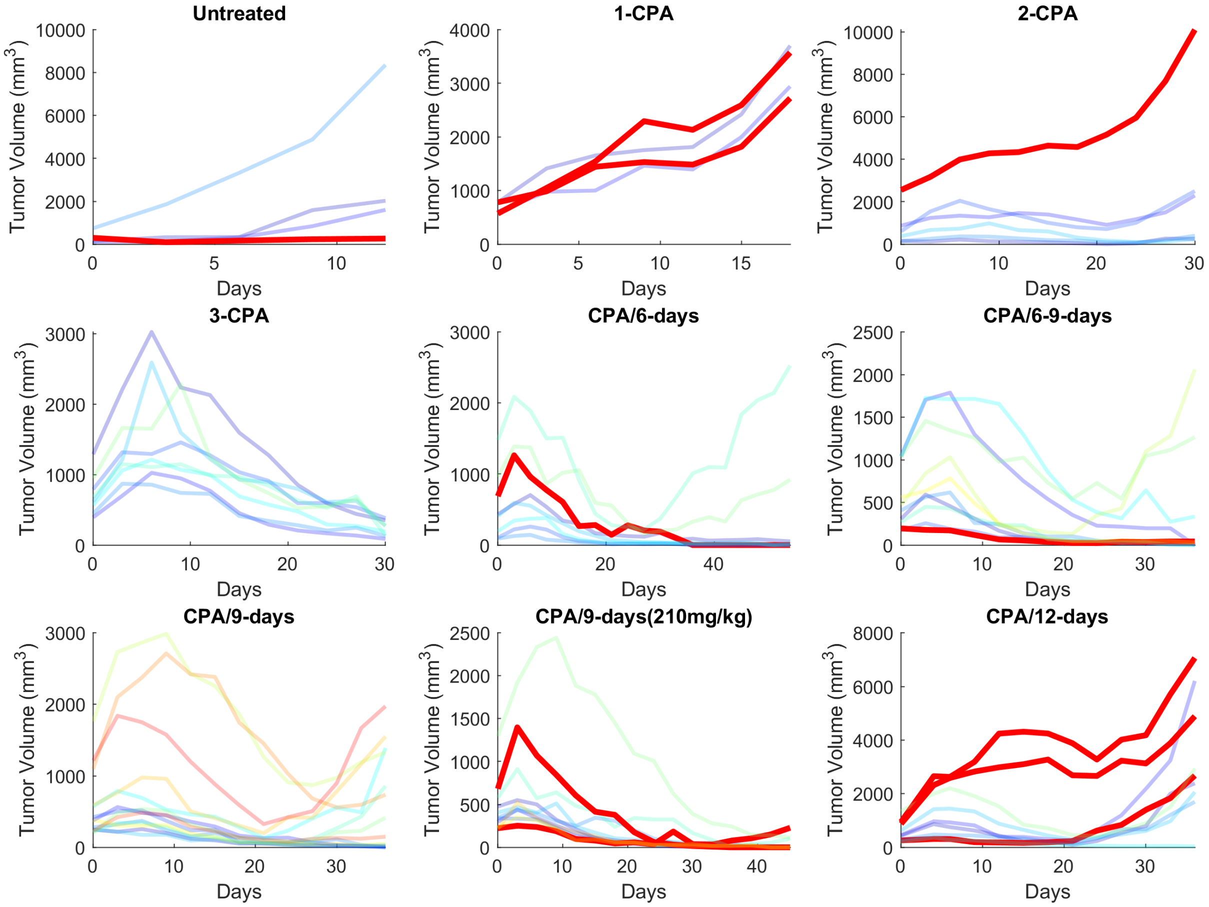

Supplementary Figure 2: The experimental data in [1] are plotted with the outliers highlighted in red. Out of the 65 time series data considering 9 different treatment conditions, 11 were excluded in the fitting of the population parameters.

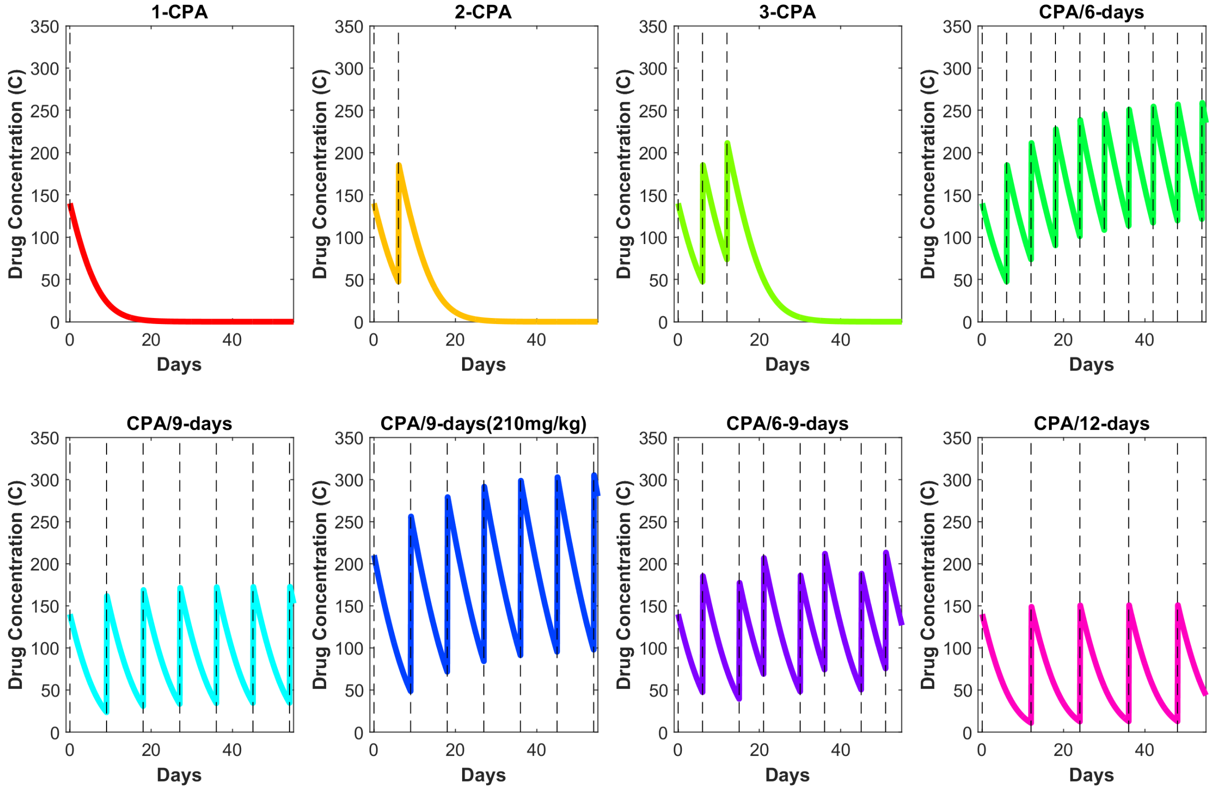

Supplementary Figure 3: Prediction of the drug concentration (C) for all the experimental conditions in [1]

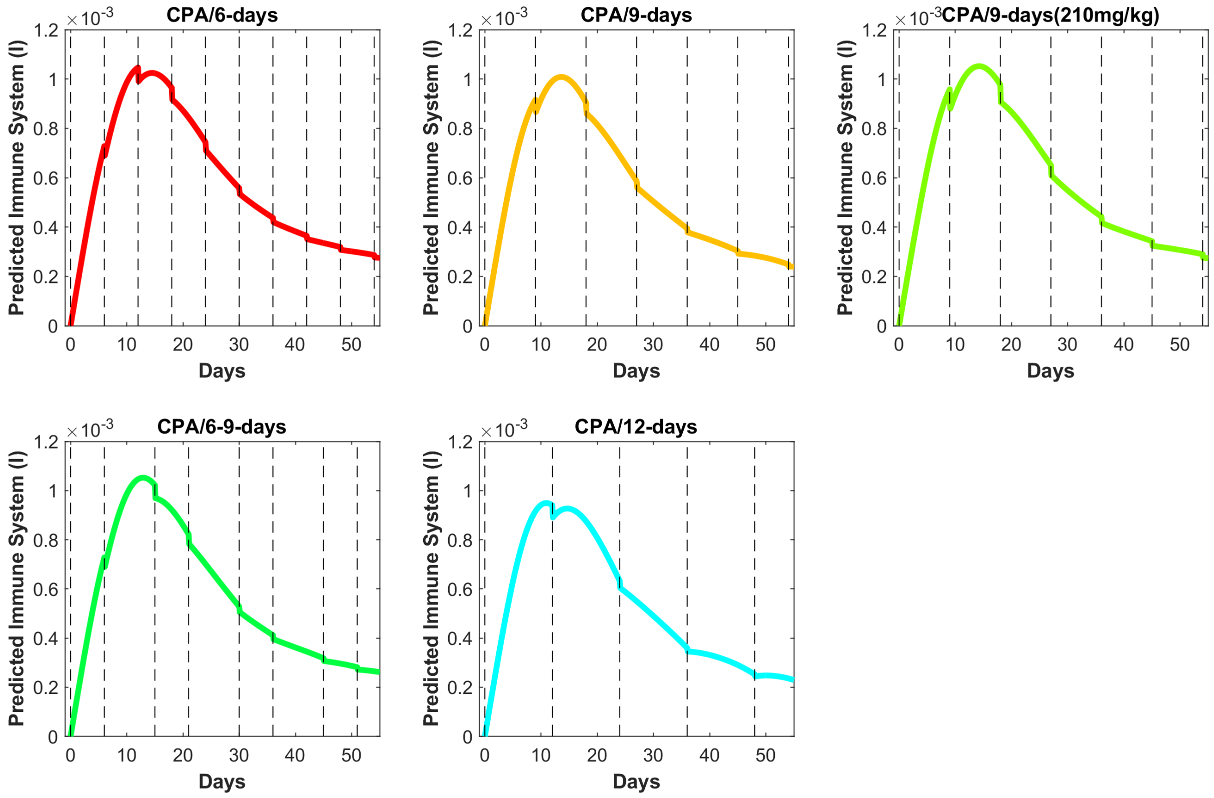

Supplementary Figure 4: Predicted immune system from the model fits for the treatment conditions other than 1-CPA, 2-CPA, and 3-CPA.

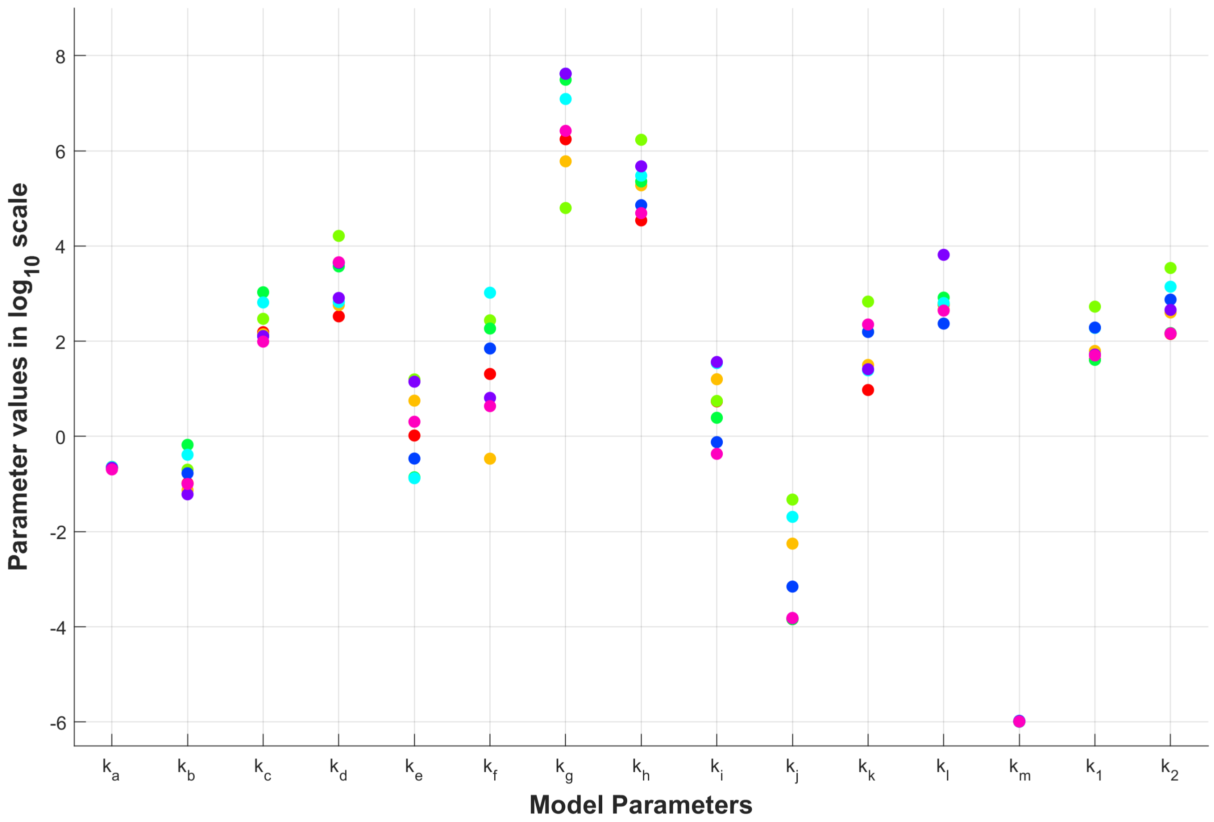

Supplementary Figure 5: Parameter values in a log_{10} scale for 8 different fits within 1% of optimal value found for the objective function. Missing dots are due to overlapping parameter values, which can be found in Supplementary Table 1.

Supplementary Table 1: Parameter values for fits within 1% of optimal value found for the objective function.

**Param.**	**Values**							
	Fit A	Fit B	Fit C	Fit D	Fit E	Fit F	Fit G	Fit H
k_a	0.2211	0.2060	0.2181	0.2180	0.2019	0.2292	0.2164	0.2270
k_b	0.1035	0.07393	0.1674	0.06062	0.1001	0.1995	0.6627	0.4101
k_c	154.8	139.7	127.7	127.4	97.83	294.7	1066	650.7
k_d	332.3	574.6	4415	809.1	4543	1.629×10^4	3723	662.9
k_e	1.042	5.621	0.3411	14.08	2.026	15.73	0.1380	0.1321
k_f	20.42	0.3397	70.21	6.443	4.312	273.9	184.3	1042
k_g	1.743×10^6	6.008×10^5	4.172×10^7	4.147×10^7	2.623×10^6	6.281×10^4	3.106×10^7	1.226×10^7
k_h	3.443×10^4	1.883×10^5	7.195×10^4	4.712×10^5	4.900×10^4	1.705×10^6	2.269×10^5	3.002×10^5
k_i	5.435	15.84	0.7552	36.49	0.4293	5.545	2.458	34.56
k_j	1.442×10^{-4}	5.571×10^{-3}	6.979×10^{-4}	1.518×10^{-4}	1.528×10^{-4}	0.04704	1.442×10^4	0.02034
k_k	9.413	31.61	156.0	25.73	223.7	679.0	157.5	24.36
k_l	585.2	461.0	233.8	6516	438.1	612.2	821.8	633.7
k_m	1.016×10^{-6}	1.015×10^{-6}	1.053×10^{-6}	1.018×10^{-6}	1.022×10^{-6}	1.027×10^{-6}	1.016×10^{-6}	1.016×10^{-6}
k_1	41.02	62.28	192.8	52.33	50.08	530.5	40.43	189.3
k_2	142.0	400.4	747.2	454.4	145.5	3450	148.6	1394

References

1. Wu, J. & Waxman, D. J. Metronomic cyclophosphamide schedule-dependence of innate immune cell recruitment and tumor regression in an implanted glioma model. *Cancer Letters* **353,** 272–280 (2014).
